# Nilotinib targets NEIL3-mediated fatty acid oxidation to promote anoikis in hepatocellular carcinoma cells

**DOI:** 10.3389/fonc.2026.1810429

**Published:** 2026-10-21

**Authors:** Biao Tang, Zhicheng Shen, Jining Zeng, Kaida Wei, Xin Tang, Min Huang, Huari He, Wangning Xia, Xintao Liu

**Affiliations:** Department of Hepatobiliary Pancreatic Spleen Surgery, The Central Hospital of Yongzhou, Yongzhou, Hunan, China

**Keywords:** anoikis, fatty acid oxidation, hepatocellular carcinoma, NEIL3, nilotinib

## Abstract

**Background:**

Overcoming anoikis is a critical step for the survival of metastatic cancer cells. Despite extensive research, resistance to anoikis in hepatocellular carcinoma (HCC) cells remains a major and yet unresolved challenge.

**Methods:**

NEIL3 expression in HCC was assessed via bioinformatics and RT-qPCR. Cell viability was measured using CCK-8. Anoikis was evaluated through live/dead staining, flow cytometry, and Western blot. Fatty acid oxidation (FAO) was evaluated by oxygen consumption rates and ATP levels. The NEIL3-Nilotinib binding was predicted by molecular docking and validated through CETSA and DARTS. Nilotinib’s inhibition of tumor growth by targeting NEIL3 was confirmed in a xenograft mouse model.

**Results:**

NEIL3 was highly expressed in HCC patient tissues and cells. NEIL3 knockdown inhibited HCC cell survival by inducing anoikis. Mechanistic studies revealed that NEIL3 was enriched in FAO pathways, and its upregulation activated FAO to suppress anoikis in HCC cells. Nilotinib specifically bound to NEIL3, which significantly counteracted inhibition of anoikis by NEIL3 overexpression and delayed tumor growth.

**Conclusion:**

By activating FAO, NEIL3 suppresses anoikis in HCC cells, a process reversible by Nilotinib through targeting NEIL3, revealing NEIL3 as a therapeutic target and Nilotinib as a candidate drug for HCC.

## 1 Introduction

The global health burden of primary liver cancer is dominated by hepatocellular carcinoma (HCC), which is both the sixth most frequently diagnosed cancer worldwide and the fourth leading cause of cancer death (1, 2). HCC pathogenesis involves a multifaceted interplay of genetic, epigenetic, and chronic liver conditions, including hepatitis and cirrhosis (3, 4). Despite progress in therapies such as local ablation, resection, and transplantation, HCC prognosis remains unfavorable owing to frequent recurrence and metastasis (5). Therefore, there is a pressing need to explore novel therapeutic targets. In recent years, integrated bioinformatics approaches have been increasingly employed to identify hub genes and potential drug targets associated with HCC pathogenesis. For instance, existing agents such as sorafenib, regorafenib, cabozantinib, and lenvatinib may serve as lead compounds for the development of novel targeted therapeutics (6). Meanwhile, emerging clinical evidence continues to reveal prognostic biomarkers for HCC. For example, Vav1 protein expression is significantly higher in HCC tumor tissues than in paired adjacent non-tumor tissues and serves as an independent unfavorable predictor of 5-year overall survival (OS), suggesting its potential utility as a molecular prognostic indicator (7). Furthermore, basic research has identified novel functional therapeutic targets in HCC: PRDX6 is upregulated in HCC, and its expression levels correlate with clinical stage and histological grade; it promotes HCC cell proliferation, migration, and invasion through activation of the PI3K/Akt/mTOR and Notch1 signaling pathways (8). Additionally, within the miR-3130-5p/FDX1 axis, upregulated miR-3130-5p promotes HCC growth in xenograft models by suppressing the tumor suppressor FDX1 (9). Collectively, these findings underscore the importance of continued molecular characterization of HCC to discover novel therapeutic targets and prognostic biomarkers. Anoikis, a programmed cell death mechanism activated upon cell detachment from the extracellular matrix, critically constrains tumor metastasis. By evading anoikis, cancer cells achieve survival advantages that drive metastatic progression and metastasis (10–12). Therefore, elucidating the regulatory mechanisms governing anoikis in HCC is essential for the development of effective therapeutic interventions aimed at improving clinical outcomes.

NEIL3, a DNA glycosylase from the NEIL family homologous to bacterial Fpg/Nei proteins (13), initiates DNA base excision repair, modulates lipid metabolism, and participates in cerebral ischemia and autoimmunity (14–18). NEIL3 also exhibits oncogenic properties. In HCC, it activates the BRAF/MEK/ERK/TWIST pathway to promote cell invasion, migration, and stemness (19). Similarly, in esophageal cancer, NEIL3 knockdown promotes apoptosis and delays tumor growth (20). Moreover, differential expression of NEIL3 influences chemotherapy resistance; reportedly, high NEIL3 expression attenuates DNA damage, promoting cisplatin resistance in lung adenocarcinoma cells (21). While these results confirm the tumor-promoting activity of NEIL3, the exact mechanisms by which it contributes to HCC development, particularly its influence on anoikis, remain unclear. Investigating the functional roles and regulatory circuits of NEIL3 in HCC is consequently vital for elucidating the disease’s molecular mechanism and discovering new treatment opportunities.

Small-molecule drugs have emerged as powerful therapeutic agents in oncology, attracting considerable research interest (22). They can translocate across the plasma membrane to interact with cell surface receptors and the cytoplasmic domains of intracellular signaling molecules. Unlike conventional cytotoxic drugs, most targeted small-molecule drugs exhibit greater specificity, reduced off-target toxicity, diminished susceptibility to resistance, enhanced selectivity, and relatively lower required doses (23, 24). As of September 2023, over 70 FDA-approved small-molecule kinase inhibitors are available for treating cancer and non-cancer indications (25). For example, gefitinib and icotinib have demonstrated significant progression-free survival benefits compared to platinum-based doublet chemotherapy (26). Nilotinib, a small-molecule tyrosine kinase inhibitor initially used for chronic myeloid leukemia (27), has recently shown potential in various solid tumors. Nilotinib can induce apoptosis and inhibit growth in tumor cells, including HCC, by suppressing multiple signaling pathways and gene expression regulation (28–30). Nevertheless, studies investigating Nilotinib’s effects in HCC are limited, and its specific mechanism of action within this context remains poorly characterized.

This work establishes that high NEIL3 expression in HCC inhibits anoikis via activation of fatty acid oxidation (FAO), promoting tumor cell survival. Furthermore, we identify the small-molecule drug Nilotinib as a direct NEIL3 binder that antagonizes its pro-tumorigenic activity. These findings elucidate a pivotal mechanistic role of NEIL3 in HCC pathogenesis and propose Nilotinib as a candidate for targeted therapy, underscoring its translational potential.

## 2 Materials and methods

### 2.1 Patient samples

10 pairs of human HCC and matched adjacent non-tumor liver tissues were obtained from patients who underwent curative surgical resection at The Central Hospital of Yongzhou. All cases were confirmed by postoperative pathological examination, and no patients had received any radiotherapy, chemotherapy, or targeted therapy prior to surgery. The use of human tissue samples in this study was approved by the Ethics Committee of The Central Hospital of Yongzhou (Approval No.: 2025122401).

### 2.2 Bioinformatic analysis

Gene expression and prognosis analysis: mRNA expression data of 50 adjacent normal tissues and 374 HCC tumor tissues were obtained from The Cancer Genome Atlas (TCGA) database. Differential expression analysis combined with literature review confirmed that NEIL3 was upregulated in HCC tissues. The association between NEIL3 expression and OS of patients in the TCGA-LIHC cohort was analyzed using the GEPIA2 database (http://gepia2.cancer-pku.cn/). Patients were divided into high- and low-expression groups using the median NEIL3 expression as the cutoff. Survival curves were plotted using the Kaplan–Meier method, and differences between groups were compared using the log-rank test, with hazard ratio (HR) and 95% confidence interval (CI) calculated.

Gene set enrichment analysis: Gene set enrichment analysis (GSEA) was performed using GSEA software (version 4.3.2) to compare the high- and low-NEIL3 expression groups. The gene sets used were the c2.cp.kegg.v2023.1.Hs.symbols and c2.cp.wikipathways.v2023.1.Hs.symbols collections from the MSigDB database. Enrichment analysis employed the default weighted enrichment statistic, and significance was assessed by permuting the phenotype labels using gene-phenotype permutation. Significantly enriched gene sets were identified at a nominal *P* value < 0.05 and a false discovery rate (FDR) < 0.25. Additionally, Pearson correlation analysis was performed to evaluate the strength of association between NEIL3 expression and pathway-related genes.

Molecular docking: The three-dimensional structure of NEIL3 protein was obtained from the RCSB PDB database (PDB ID: 7JL5), and the small-molecule ligand library was retrieved from the DrugBank database (version 5.1.10). Prior to docking, the protein structure was pretreated using AutoDockTools (version 1.5.7) to add hydrogen atoms and charges, remove water molecules, and merge non-polar hydrogens. Docking calculations were performed in a semi-flexible docking mode, with the protein set as rigid and the ligand set as flexible. The search space (grid box) covered the entire surface of the protein, with a grid spacing of 1.0 Å. The scoring function was the empirical potential-based function implemented in AutoDock Vina, and binding free energy was used as the screening metric. For each small molecule, the top 10 binding conformations with the lowest binding free energy were output. Candidate compounds were selected based on binding affinity and the rationality of the binding mode, and the small molecule with the lowest binding free energy and the most stable binding mode was chosen as the lead compound. To further elucidate the binding mode between the lead compound and NEIL3, PyMOL software (version 2.5) and LigPlot+ software (version 2.2) were used to generate three-dimensional and two-dimensional interaction diagrams, respectively, to analyze hydrogen bonds and hydrophobic interactions between them.

### 2.3 Cell cultivation

The study obtained human normal liver epithelial cells THLE-3 (CRL-3583) and HCC cell lines SNU-475 (CRL-2236) and Hep3B (HB-8064) from ATCC (USA) and HCC Huh-7 cells SCSP-526 from the National Collection of Authenticated Cell Cultures (China). Cells were cultivated in the following media supplemented with 10% FBS (Gibco) and 1% penicillin-streptomycin: THLE-3 in BEGM BulletKit (Lonza, CC-3170, Switzerland); SNU-475 in RPMI-1640 (ATCC, 30-2001, USA); Hep3B in EMEM (ATCC, 30-2003, USA); Huh-7 in DMEM (Gibco, 11995065, USA). For specific experiments, cells were treated with 40 μM Etomoxir (Eto, MCE, HY-50202, USA) for 4 h (31).

### 2.4 Lentiviral and plasmid transfection

shRNA lentiviral particles targeting NEIL3 (sh-NEIL3), a pcDNA3.1 NEIL3 overexpression plasmid (oe-NEIL3), and controls were from GenePharma (China). For lentiviral transduction, viral particles were added to culture medium containing Polybrene (Absin, abs42025397, China) to enhance virus-cell binding and transduction efficiency. Overexpression plasmids were transfected into cells with Lipofectamine 3000 (Invitrogen, USA). Transfection efficiency was evaluated by RT-qPCR. The target sequences used for NEIL3 knockdown were sh-NEIL3-F (5′-CGGATTCATTTCGGAATGAAA-3′) and sh-NEIL3-R (5′-TTTCATTCCGAAATGAATCCG-3′).

### 2.5 RT-qPCR

Isolated with VeZol Reagent (Vazyme, R411-01, China), total RNA was reverse-transcribed with the HiScript III 1st Strand cDNA Synthesis Kit (Vazyme, R312-01, China). qPCR was performed with ChamQ Universal SYBR qPCR Master Mix (Vazyme, Q711-02, China) on a 7500 Fast Real-Time PCR System. Relative expression was calculated via the 2^-ΔΔCt^ method, normalized to β-actin.

Primers (5’-3’): NEIL3-F: TCTCCTGTTTTGGAAGTGCAG; NEIL3-R: CATTAGCACATCACCTAGCATC; β-actin-F: CTGGAACGGTGAAGGTGACA; β-actin-R: GTCCTCGGCCACATTGTGAA.

### 2.6 CCK-8 assay

Cells were seeded in poly-HEMA (Sigma-Aldrich, 529265, USA)-coated 96-well plates. Viability was assessed at 0, 24, 48, and 72 h with a CCK-8 kit (Beyotime, C0041, China) by adding 10 μL/well CCK-8 working solution and measuring optical density (OD) values at 450 nm.

### 2.7 Western blot (WB)

Total protein was extracted by lysing cells in RIPA lysis buffer (Sangon Biotech, China). Protein concentration was quantified with a BCA protein assay kit (Beyotime, P0010, China), normalized across samples, mixed with 5× SDS-PAGE loading buffer (Solarbio, China), and boiled for denaturation. Proteins were separated by SDS-PAGE and transferred onto PVDF membranes (Millipore, USA). Membranes were blocked with 5% skim milk for 1 h, washed with TBST (Servicebio, China), and incubated overnight at 4 °C with primary antibodies against cleaved-PARP (ab32064), cleaved-caspase3 (ab32042), NEIL3 (ab110049), or β-actin (ab8226; all from Abcam, UK). Membranes were then probed with the HRP-conjugated secondary antibody (Abcam, UK) for 1 h at room temperature. After a final wash, immunoreactive bands were detected with ECL reagent (Proteintech, USA) and imaged with a chemiluminescence detection system (Clinx, China).

### 2.8 Anoikis assay

For live/dead staining, cells (1×10^5^/well) were cultured in poly-HEMA-coated 24-well plates for 24h, rinsed with 1× PBS three times, stained with Calcein AM/EthD-I (Proteintech, PF00008, USA) in the dark, and imaged by fluorescence microscopy. For apoptosis detection, cells (1×10^5^/well) cultured in poly-HEMA-coated 6-well plates for 24 h were stained with Annexin V-FITC/PI (15 min) using an apoptosis detection kit (MCE, HY-K1073, USA) and analyzed by flow cytometry (Agilent, USA).

### 2.9 Oxygen consumption rate (OCR)

After 24 h cultivation on poly-HEMA-coated plates, floating cell spheroids were collected, gently centrifuged (300×g, 5 min), and resuspended in serum-free medium containing 0.05% Cell-Tak (BD, USA). The suspension was seeded into pre-coated Seahorse assay plates, centrifuged (300×g, 5 min), and allowed to adhere for 1 h at room temperature. OCR was measured with a Seahorse XFe 96 analyzer (Agilent, USA). Briefly, OCR was recorded after calibration for the basal value, and sequential injections of Oligomycin, FCCP, and Rotenone/Antimycin A (all from MCE, USA) to monitor OCR changes.

### 2.10 ATP, acetyl-CoA, and free fatty acid (FFA) levels

ATP assay: Following kit instructions (Solarbio, BC0305, China), cell lysates were centrifuged (10,000×g, 10 min, 4 °C). The supernatant was mixed with 500 μL chloroform and centrifuged again (10,000 × g, 3 min, 4 °C). Subsequent reagents were added per the protocol. OD at 340 nm was measured and used to calculate ATP concentration according to the kit formula.

Acetyl-CoA assay: The acetyl-CoA content in cells of each group was measured using an enzyme-linked immunosorbent assay (ELISA) with a human acetyl-CoA ELISA kit (CB12747-Hu, Coibo Bio, China). Cells from each group were collected, washed twice with pre‑chilled PBS, and then lysed. After centrifugation at 10,000 × g for 15 minutes at 4 °C, the supernatant was collected as the test sample. The assay was performed following the manufacturer’s instructions, and the absorbance was measured at 450 nm. The acetyl-CoA concentration in each sample was calculated based on the standard curve and normalized to that of the control group.

FFA assay: The FFA content in cells of each group was measured using an ELISA with a human FFA ELISA kit (CB11693-Hu, Coibo Bio, China). Cells from each group were collected and lysed with lysis buffer to extract total lipids. After centrifugation, the supernatant was collected as the test sample. The assay was performed following the manufacturer’s instructions, and the absorbance was measured at 450 nm. The FFA concentration in each sample was calculated based on the standard curve and normalized to that of the control group.

### 2.11 Cellular thermal shift assay (CETSA)

Cells treated with Nilotinib (Selleck, S1033, USA) or DMSO for 24 h were resuspended in lysis buffer, heated in a water bath at graded temperatures (45-80 °C), snap-cooled in a nitrogen canister, and lysed. The supernatant was collected for WB analysis.

### 2.12 Drug affinity responsive target stability (DARTS)

Protein extracts from cell lysates were incubated with Nilotinib or DMSO for 3 h at room temperature and subjected to hydrolysis with Pronase E (Merck, Germany). Digestion was terminated by boiling at 95 °C for 10 min before subsequent WB.

### 2.13 Xenograft mouse model

Twenty-four female BALB/c nude mice (5–6 weeks old) purchased from Beijing Vital River Laboratory Animal Technology Co., Ltd. (China) were housed under a 12-h light/dark cycle with free access to food and water. After one week of acclimation, nude mice were randomly allocated into the oe-NC+PBS, oe-NEIL3+PBS, oe-NC+Nilotinib, or oe-NEIL3+Nilotinib group (n=6). They were subcutaneously injected with SNU-475 cells stably expressing oe-NC or oe-NEIL3. Once tumors reached 100 mm^3^, mice received 20 mg/kg Nilotinib (32) or PBS via oral gavage every two days. Tumors were measured regularly (volume = length×width^2^/2). After 25 days, mice were euthanized, and tumors were harvested. All procedures were approved by The Central Hospital of Yongzhou Animal Care and Use Committee (Approval No.: 2025122401).

### 2.14 Immunohistochemistry (IHC)

After baking, deparaffinization, and rehydration, paraffin-embedded tumor sections (5 μm) underwent antigen retrieval in boiling Tris-EDTA buffer (1 mmol/L, pH 9.0), endogenous peroxidase blocking with 3% H_2_O_2_ (Sigma-Aldrich, USA), and 5% BSA blocking (Sigma-Aldrich, USA). Sections were incubated with primary antibodies (overnight, 4 °C; anti-NEIL3, HUABIO, ER64147, China; anti-Ki67 Abcam, ab16667, UK; anti-cleaved caspase-3, Proteintech, 25128-1-AP, USA; or anti-CPT1A, Abcam, ab128568, UK) and with secondary antibodies (Abcam, UK) at 37 °C for 30 min. Sections were stained with DAB (Proteintech, USA) and hematoxylin (Beyotime, China), dehydrated, cleared, mounted with neutral balsam, and imaged under a microscope.

### 2.15 Statistical analysis

Data are presented as mean ± SD from at least three independent experiments and were analyzed in GraphPad 8.0. Statistical significance (*P* < 0.05) was determined by Student’s *t*-test for two groups or one-way ANOVA with Tukey’s *post-hoc* test for multiple groups.

## 3 Results

### 3.1 NEIL3 knockdown induces anoikis in HCC cells

Bioinformatics and literature review identified NEIL3 as a candidate mRNA. Wilcoxon test revealed significantly higher NEIL3 expression in HCC tissues compared to adjacent normal tissues (**Figure 1A**). In this study, 10 pairs of HCC and matched adjacent non‑tumor tissue samples were collected, and the mRNA levels of NEIL3 were detected by RT‑qPCR. The results showed that NEIL3 was significantly upregulated in HCC tissues compared with paired adjacent non‑tumor tissues (**Figure 1B**). RT‑qPCR confirmed NEIL3 overexpression in multiple HCC cell lines versus normal human liver epithelial cells (**Figure 1C**). To further validate the association between NEIL3 expression and the prognosis of HCC patients, we analyzed the relationship between NEIL3 expression and patient prognosis in the TCGA‑LIHC cohort using the GEPIA2 database. Kaplan–Meier survival curves demonstrated that patients in the high NEIL3 expression group had significantly shorter OS than those in the low expression group (**Figure 1D**). GSEA indicated a significant positive correlation between NEIL3 expression and the anoikis signaling pathway (**Figure 1E**), suggesting the involvement of NEIL3 in anoikis regulation in HCC. NEIL3 functions were investigated in stable NEIL3-knockdown Huh-7 cells and controls, with RT-qPCR confirming efficient knockdown (**Figure 1F**). CCK-8 assays showed that NEIL3 knockdown significantly reduced Huh-7 cell proliferation (**Figure 1G**). Anoikis was induced through suspension cell culture with poly-HEMA-coated plates. Live/dead staining demonstrated increased red fluorescence in Huh-7 cells upon NEIL3 knockdown, indicating enhanced anoikis (**Figure 1H**). Annexin V/PI flow cytometry confirmed a significant increase in apoptosis under suspension culture conditions in NEIL3-knockdown cells (**Figure 1I**). WB analysis showed upregulation of the apoptosis markers cleaved-PARP and cleaved-caspase3 (**Figure 1J**). In conclusion, NEIL3 overexpression in HCC promotes cancer cell survival by suppressing anoikis.

**Figure 1:**
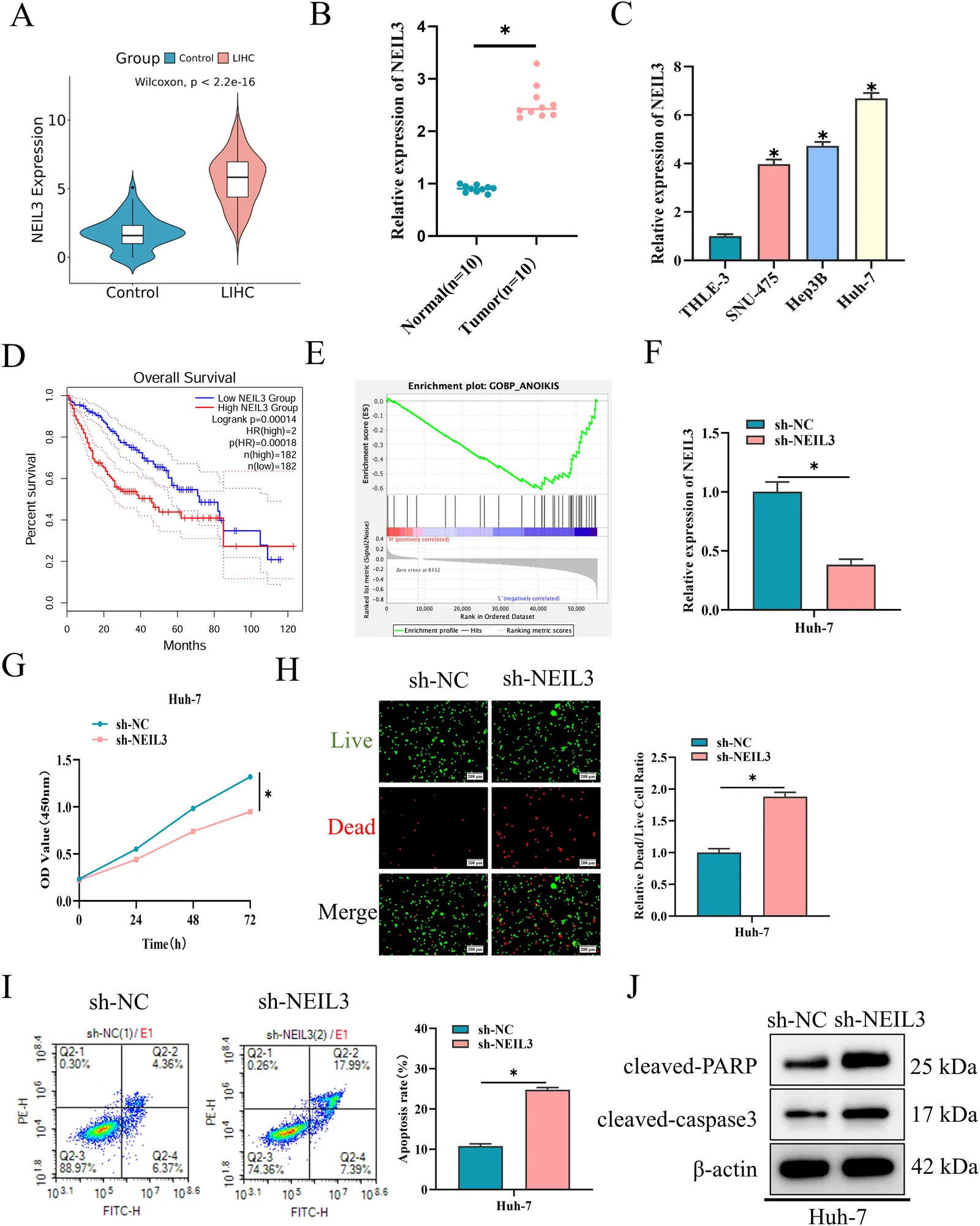
NEIL3 knockdown induces anoikis in HCC cells. **(A)** NEIL3 mRNA expression in normal adjacent and HCC tumor tissues from the TCGA database; differential expression analyzed by the Wilcoxon test. **(B)** RT-qPCR analysis of NEIL3 in HCC tumors and their paired adjacent normal tissues. **(C)** RT-qPCR analysis of NEIL3 mRNA expression in the normal human liver epithelial cell line (THLE-3) and HCC cell lines (SNU-475, Hep3B, Huh-7). **(D)** Analysis of the relationship between different NEIL3 expression levels and OS of HCC patients in the TCGA-LIHC cohort using the GEPIA2 database. **(E)** GSEA showing enrichment pathways of NEIL3. Huh-7 cells were infected with sh-NEIL3 or control lentivirus. **(F)** RT-qPCR validation of NEIL3 knockdown efficiency. Huh-7 cells infected with sh-NEIL3 or control lentivirus were cultured in poly-HEMA-coated plates for 24 h. **(G)** Cell viability measured by CCK-8 assay. **(H)** Representative fluorescence images from live/dead staining. **(I)** Apoptosis analysis by Annexin V/PI staining and flow cytometry. **(J)** Western blot analysis of cleaved-PARP and cleaved-caspase3 expression. All quantitative data are presented as the mean ± standard deviation from three independent experiments (n = 3). Comparisons between two groups were performed using unpaired Student’s t‑test **(B, F–I)**, and comparisons among multiple groups were performed using one‑way analysis of variance (ANOVA) followed by Tukey’s *post hoc* test **(C)**. *p < 0.05.

### 3.2 NEIL3 suppresses anoikis in HCC by activating FAO

To explore the potential mechanism underlying the anti‑apoptotic function of NEIL3, we performed GSEA to identify potential pathways associated with NEIL3 expression in HCC. The results revealed enrichment of NEIL3 in fatty acid metabolism and oxidation pathways (**Figures 2A, B**). To determine representative candidate genes for subsequent correlation analysis, we first ranked the fatty acid metabolism‑related gene sets enriched by GSEA according to their correlation with NEIL3 expression. Based on the functional complementarity of these genes in FAO pathways, we selected genes involved in four key nodes: substrate hydrolysis (PPT1) (33), cytosolic acyl‑CoA activation (ACSL4, ACSL3) (34), and peroxisomal transport (ABCD1) (35). Pearson correlation analysis showed that NEIL3 was significantly positively correlated with all four key FAO genes (**Figure 2C**). Given that FAO inhibits anoikis, we overexpressed NEIL3 in SNU‑475 cells with or without the FAO inhibitor etomoxir (Eto). NEIL3 overexpression significantly increased the oxygen consumption rate, ATP levels, and acetyl‑CoA levels, while decreasing FFA content (**Figures 2D, E**), suggesting enhanced FAO activity. Moreover, NEIL3 overexpression enhanced cell viability (**Figure 2F**), reduced cells with red fluorescence (**Figures 2G, H**), and decreased apoptosis (**Figures 2I–K**). Inhibition of FAO with Eto reversed these effects, restoring SNU-475 cell viability and apoptosis levels to those of the control group (**Figures 2F–K**). In summary, NEIL3 suppresses anoikis in HCC by activating FAO.

**Figure 2:**
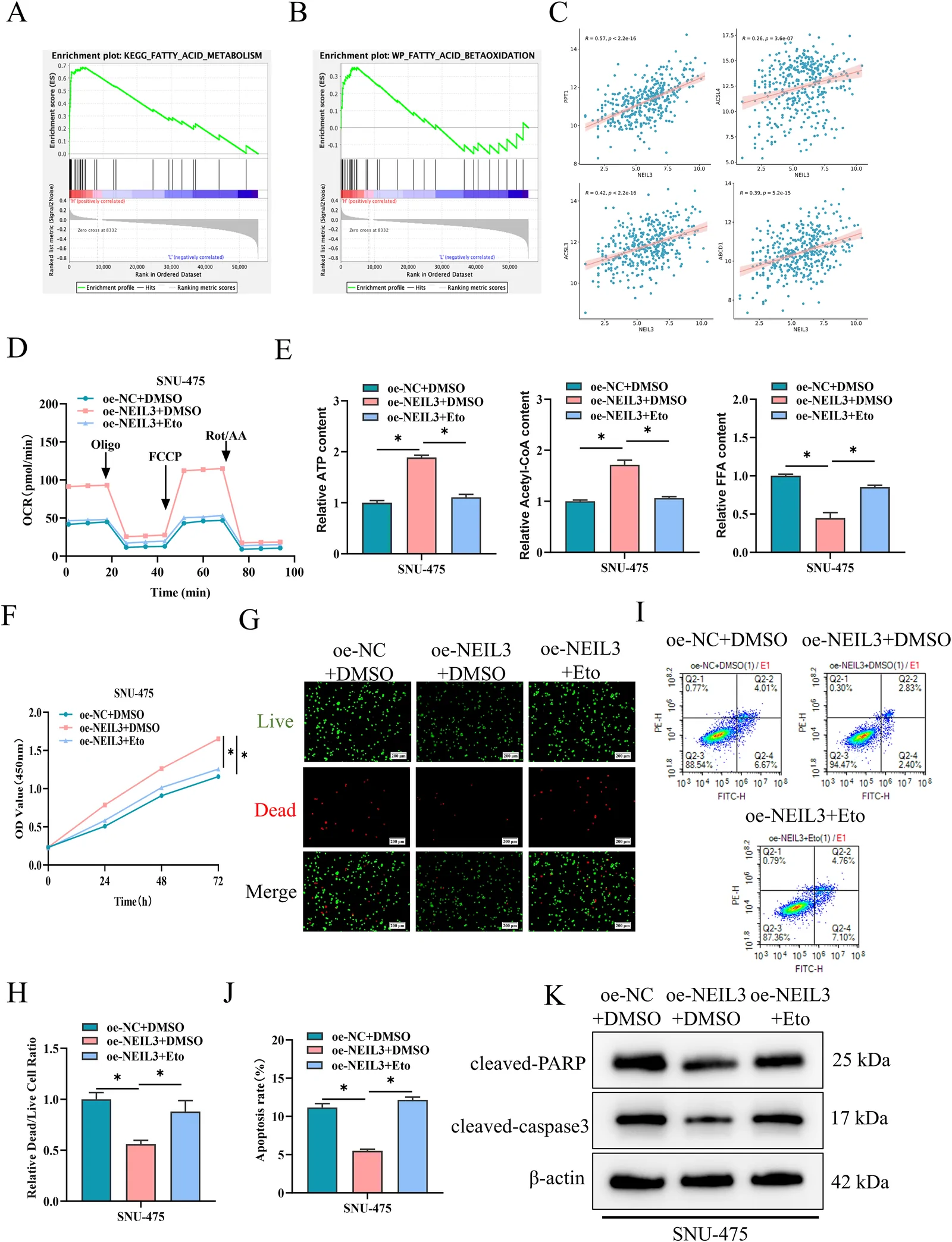
NEIL3 suppresses anoikis in HCC by activating FAO. **(A, B)** GSEA showing enrichment of NEIL3 in fatty acid metabolism **(A)** and oxidation **(B)** pathways. **(C)** Pearson correlation analysis between NEIL3 and key FAO-related genes (PPT1, ACSL4, ACSL3, ABCD1). SNU-475 cells transfected with oe-NEIL3 or control plasmid (oe-NC) were treated with or without 40 µM Etomoxir (Eto) for 4 h, then cultured on poly-HEMA-coated plates for 24 h. **(D)** Oxygen consumption rate (OCR) measured by Seahorse XFe96 analyzer. **(E)** The relative levels of ATP, acetyl-CoA, and free fatty acids (FFA) in each group were determined using biochemical assay kits. **(F)** Cell viability assessed by CCK-8 assay. **(G, H)** Representative images **(G)** and quantification **(H)** from live/dead staining. **(I, J)** Representative flow cytometry plots **(I)** and quantification **(J)** of apoptosis by Annexin V/PI staining. **(K)** Western blot analysis of cleaved-PARP and cleaved-caspase3. All quantitative data are presented as the mean ± standard deviation from three independent experiments (n = 3). Comparisons among multiple groups were performed using one‑way analysis of variance (ANOVA) followed by Tukey’s *post hoc* test **(D–F, H, J)**. *p < 0.05.

### 3.3 Nilotinib targets NEIL3

Based on our previous findings that NEIL3 promotes cell survival through the activation of FAO, we further sought to screen for small-molecule compounds capable of targeting NEIL3 and blocking its function, with the aim of exploring potential therapeutic strategies for HCC. Batch molecular docking against the NEIL3 protein was performed to screen for potential binders. Nilotinib was identified as the top candidate with the lowest binding affinity score. Interaction analysis revealed that Nilotinib forms hydrophobic interactions with NEIL3 residues Arg175, Asp183, Gln184, and Asn192, and hydrogen bonds with Lys81 and Arg271 (2D and 3D interaction diagrams in **Figures 3A, B**). CETSA and the DARTS assay validated such binding in Huh-7 cells. In the CETSA, Huh-7 cells were incubated with or without Nilotinib and heated at varying temperatures. Assessed by WB, Nilotinib treatment increased NEIL3 protein stability upon thermal challenge compared to the DMSO control (**Figure 3C**), indicating that Nilotinib stabilizes NEIL3 protein. In the DARTS assay, Huh-7 cell lysates were incubated with varying concentrations of Nilotinib or vehicle control for 3 h, followed by Pronase E treatment. Results indicated that Nilotinib protected NEIL3 from Pronase E digestion in a concentration-dependent manner (**Figure 3D**). Collectively, these data confirm the specific binding of Nilotinib to NEIL3.

**Figure 3:**
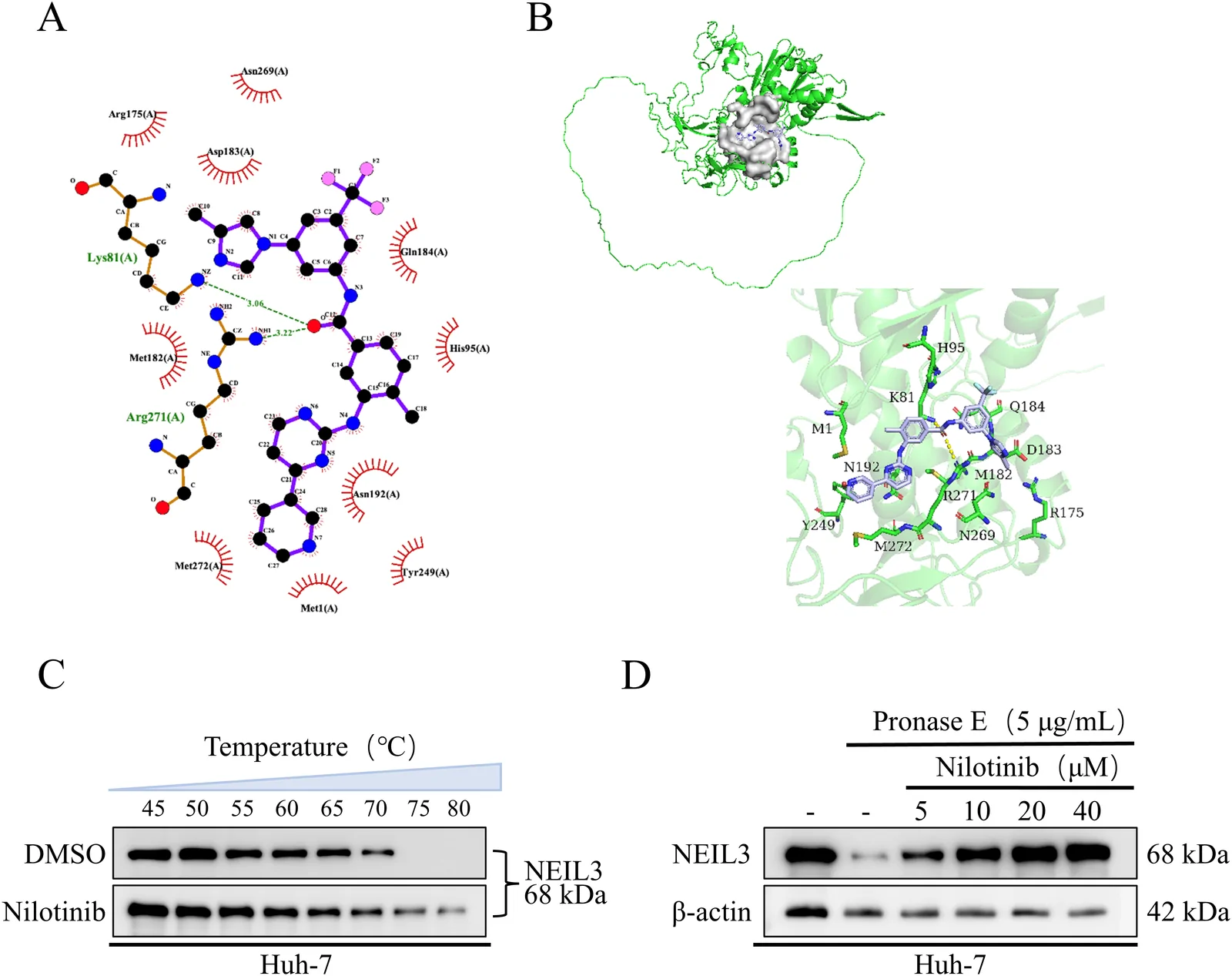
Nilotinib targets NEIL3. **(A, B)** Two-dimensional **(A)** and three-dimensional **(B)** diagrams of molecular docking between Nilotinib and NEIL3. **(C)** CETSA validation of Nilotinib binding to NEIL3 using Huh-7 cells treated with 20 µM Nilotinib or DMSO for 24 h. **(D)** DARTS validation of binding using protein extracts from Huh-7 cells incubated with the indicated concentrations of Nilotinib for 3 h, followed by Pronase E digestion.

### 3.4 Nilotinib promotes anoikis in HCC by targeting NEIL3

We next investigated whether Nilotinib affects HCC anoikis by targeting NEIL3. SNU-475 cells were divided into four groups: oe-NC+DMSO, oe-NEIL3+DMSO, oe-NC+Nilotinib, and oe-NEIL3+Nilotinib. After 24 h of culture, OCR analysis revealed a significant decrease in the oe-NC+Nilotinib group compared to the oe-NC+DMSO control. Furthermore, OCR in the oe-NEIL3+Nilotinib group was notably higher than that in the oe-NC+Nilotinib group (**Figure 4A**). Similarly, NEIL3 overexpression increased cellular ATP levels, which was counteracted by Nilotinib (**Figure 4B**). The CCK-8 assay confirmed that Nilotinib inhibited the pro-viability effect of NEIL3 (**Figure 4C**). Live/dead staining and flow cytometry analyses demonstrated that Nilotinib treatment reversed the suppression of anoikis by NEIL3 overexpression (**Figures 4D, E**). These findings indicate that Nilotinib promotes anoikis in HCC cells by antagonizing NEIL3-mediated activation of FAO.

**Figure 4:**
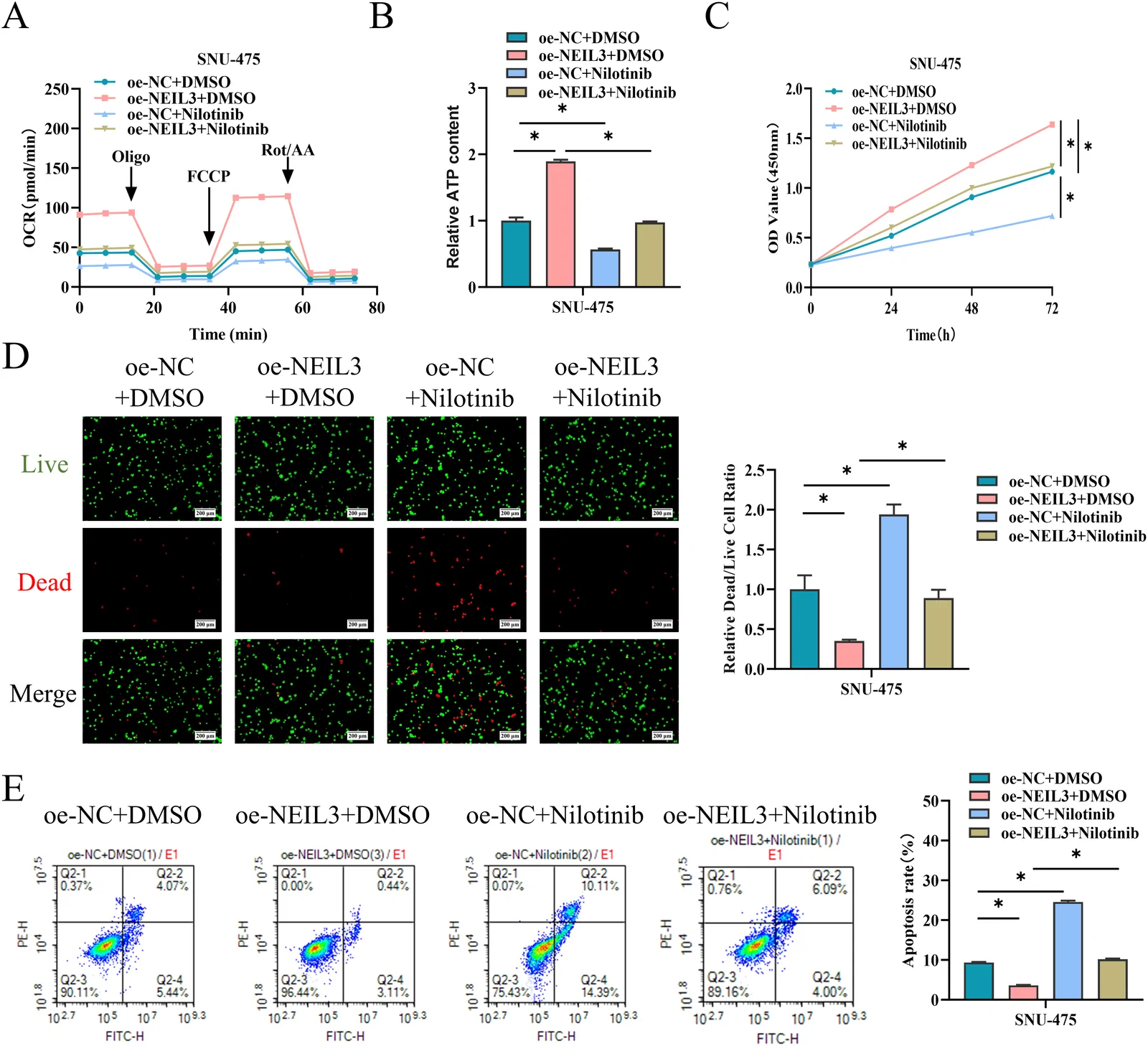
Nilotinib promotes anoikis in HCC by targeting NEIL3. SNU-475 cells transfected with oe-NEIL3 or control plasmid (oe-NC) were treated with 20 µM Nilotinib or DMSO for 24 h, then cultured on poly-HEMA-coated plates for 24 h. **(A)** Oxygen consumption rate (OCR) measured by a Seahorse XFe96 Analyzer. **(B)** Cellular ATP levels determined using a commercial assay kit. **(C)** Cell viability assessed by CCK-8 assay. **(D)** Fluorescence microscopy of cells stained with a live/dead kit. **(E)** Apoptosis analyzed by flow cytometry. All quantitative data are presented as the mean ± standard deviation from three independent experiments (n = 3). Comparisons among multiple groups were performed using one‑way analysis of variance (ANOVA) followed by Tukey’s *post hoc* test **(A–E)**. *p < 0.05.

### 3.5 Nilotinib inhibits HCC growth by binding to NEIL3 *in vivo*

To evaluate the therapeutic effect of Nilotinib via NEIL3 targeting *in vivo*, nude mice bearing subcutaneous xenografts of SNU-475 cells expressing oe-NC or oe-NEIL3 were treated with 20 mg/kg Nilotinib or PBS. Compared to the oe-NC+PBS group, the oe-NEIL3+PBS group exhibited significantly increased tumor volume and weight. Nilotinib treatment markedly suppressed this NEIL3 overexpression-driven tumor growth and weight (**Figures 5A–C**), indicating that Nilotinib effectively suppressed HCC tumor growth mediated by NEIL3 overexpression. IHC analysis revealed that tumors from the oe-NEIL3+PBS group had higher expression of Ki67 and lower expression of cleaved-caspase3 compared to the oe-NC+PBS group. Nilotinib treatment significantly reduced Ki67 expression and increased cleaved-caspase3 levels (**Figure 5D**), indicating that Nilotinib can rescue the effects of NEIL3 overexpression on cell apoptosis and proliferation ability. Expression of the fatty oxidation marker CPT1A was significantly higher in the oe-NEIL3+PBS group but reduced by Nilotinib treatment (**Figure 5D**). Notably, Nilotinib did not affect NEIL3 protein expression itself (**Figure 5D**). However, by binding to NEIL3, Nilotinib suppresses FAO and promotes HCC cell apoptosis, thereby inhibiting tumor growth.

**Figure 5:**
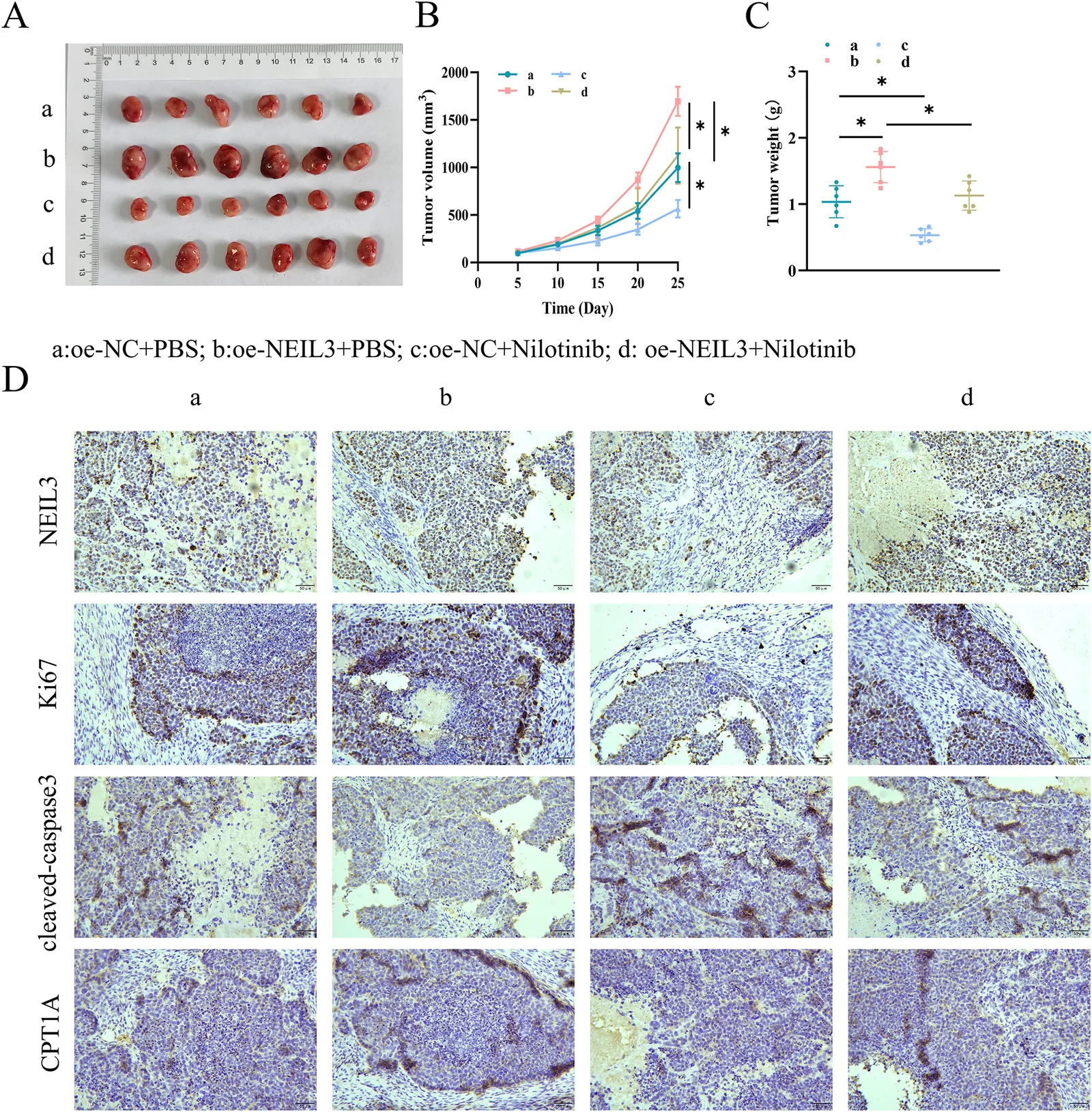
Nilotinib inhibits HCC growth by binding NEIL3 *in vivo*. Nude mice were subcutaneously injected with SNU-475 cells stably expressing oe-NC or oe-NEIL3. When tumor volume reached 100 mm^3^, mice were treated with 20 mg/kg Nilotinib or PBS. Nude mice were euthanized on day 25. **(A, B)** Tumor volume in nude mice: representative images **(A)** and quantification **(B)**. **(C)** Tumor weights at endpoint. **(D)** Immunohistochemical images of NEIL3, Ki67, cleaved-caspase3, and CPT1A expression in tumor tissues. All quantitative data are presented as the mean ± standard deviation (n = 6 nude mice per group). Comparisons among multiple groups were performed using one‑way analysis of variance (ANOVA) followed by Tukey’s *post hoc* test **(B, C)**. *p < 0.05.

## 4 Discussion

High metastatic propensity is a major contributor to the poor prognosis of HCC patients (36, 37). The metastatic cascade requires detached tumor cells to survive in the circulation and eventually colonize distant sites. The ability to survive detachment from the extracellular matrix is therefore crucial, and anoikis is a key programmed cell death mechanism regulating this survival (38, 39). However, the molecular mechanisms underlying anoikis resistance in HCC and corresponding therapeutic strategies remain incompletely understood, presenting a significant challenge. In this study, we discovered for the first time that NEIL3 suppresses anoikis in HCC cells and elucidated the mechanism of Nilotinib impeding HCC progression by targeting NEIL3. By clarifying the regulatory relationship between NEIL3 and Nilotinib, we provide a novel target and therapeutic strategy for overcoming anoikis resistance in HCC.

As a DNA glycosylase, NEIL3 primarily functions to preserve telomere integrity, such as by repairing oxidative damage to telomeres during mitosis, and to maintain overall genomic stability. Demonstrated by Zhao et al. (40), NEIL3 repairs oxidative damage by recruiting APE1 to telomeric damage sites in HCC. NEIL3 knockdown leads to telomere fusion and chromosomal instability, and its expression correlates negatively with TNM stage and OS/PFS (40, 41). *In vitro* studies have also implicated high NEIL3 expression in regulating the HCC cell cycle, proliferation, apoptosis, and epithelial-mesenchymal transition (19, 42). Furthermore, NEIL3 activates metabolic pathways, such as aerobic glycolysis and glutamine metabolism, to promote angiogenesis and macrophage M2 polarization in HCC (43, 44). These findings suggest that NEIL3 is a multifunctional regulatory node linking metabolic reprogramming and tumor progression, but its role in the regulation of anoikis has not yet been explored.

As a DNA glycosylase, NEIL3 has a known function in base excision repair at gene regulatory elements (45). Its overexpression alleviates oxidative stress and effectively repairs oxidative DNA damage induced by reactive oxygen species (ROS) (46, 47). Notably, oxidative stress inhibits the transcriptional activity of PPARα (48), a key transcription factor that regulates FAO and upregulates the expression of key enzymes such as CPT1A (49). CPT1A, the rate‑limiting enzyme of FAO, has been demonstrated to mediate anoikis resistance in various tumor types (50). Therefore, by efficiently repairing oxidative damage and alleviating oxidative stress, NEIL3 overexpression may indirectly relieve the inhibition of PPARα, thereby maintaining or even enhancing the transcription of FAO‑related genes. This upregulation of FAO is particularly important under anoikis‑prone conditions, as tumor cells deprived of extracellular matrix typically exhibit suppressed glucose uptake and catabolism, leading to depletion of ATP and NADPH. In this context, activation of FAO provides ATP to maintain cellular energy homeostasis, preventing energy crisis‑induced anoikis (51). Accordingly, targeting FAO has been shown to effectively promote anoikis and inhibit tumor progression in multiple tumor types. For example, in colorectal cancer, CPT1A‑mediated FAO activation resists anoikis by scavenging ROS, and its high expression is closely associated with the formation of clinical metastases (52). In ovarian cancer, targeting FAO has been demonstrated to effectively promote anoikis and inhibit tumor progression (53). In esophageal squamous cell carcinoma, CPT1A is significantly upregulated under detachment conditions and promotes anoikis resistance by maintaining redox homeostasis (50). Collectively, these lines of evidence indicate that FAO activation represents a common survival strategy for tumor cells to resist anoikis. Our experimental results are highly consistent with this paradigm: NEIL3 was enriched in fatty acid metabolism and oxidation pathways, and its overexpression led to elevated acetyl‑CoA levels, decreased FFA content, accompanied by increases in oxygen consumption rate and ATP production, indicating enhanced FAO activity. More importantly, when FAO was blocked by the inhibitor etomoxir, both the metabolic effects and the anti‑anoikis function were reversed, directly supporting the functional mechanism by which NEIL3 promotes anoikis resistance through activation of the FAO pathway. Based on the above literature evidence and experimental validation, this study establishes the existence of the NEIL3‑FAO‑anoikis resistance axis.

These findings collectively establish NEIL3 as a significant contributor to HCC pathogenesis. Extending this knowledge, we now identify a novel role for NEIL3 in tumor anoikis, demonstrating that its elevated expression inhibits anoikis in HCC cells. Given the centrality of metabolic reprogramming in cancer (54) and NEIL3’s connection to tumor metabolism, our analysis reveals a specific functional enrichment of NEIL3 in fatty acid metabolism and oxidation. Crucially, prior studies demonstrate that activation of FAO can confer resistance to anoikis in cancer cells (52, 53, 55). Our results confirm the presence of the NEIL3-FAO-anoikis resistance axis. These insights advance the mechanistic understanding of NEIL3 in HCC and reveal a novel potential point of therapeutic intervention.

In recent years, drug development has increasingly focused on targeting specific tumor molecules, with small-molecule drugs being a prominent class. Identifying novel and effective small-molecule drugs targeting HCC has become a critical focus in HCC therapy (56). In this work, we performed a targeted drug screen against the NEIL3 protein and, for the first time, identified Nilotinib as a binding partner. As a second-generation BCR-ABL tyrosine kinase inhibitor, Nilotinib is approved for treating adult and pediatric chronic myeloid leukemia, primarily by blocking BCR-ABL-driven leukemic cell proliferation (57, 58). Nilotinib also shows application potential in solid tumors. For example, by inhibiting the activity of ABC transporters like ABCC10/MRP7, Nilotinib can overcome multidrug resistance and restore cancer cell sensitivity to specific chemotherapeutics (59). Therapeutic activity of Nilotinib has also been observed across melanoma, colorectal cancer, and HCC (29, 30, 60). Clinical studies suggest that combining Nilotinib with chemotherapeutic drugs such as paclitaxel can significantly enhance antitumor effects (61). Therefore, a deeper investigation into the anticancer efficacy of Nilotinib represents a promising strategy to address the current therapeutic challenges in HCC. Our integrated *in vitro* and *in vivo* analyses confirm that Nilotinib targets the NEIL3-FAO axis, promoting anoikis and inhibiting HCC tumor growth. These findings provide compelling evidence and a scientific rationale for exploring Nilotinib in HCC treatment.

In summary, our work elucidates a mechanism through which Nilotinib inhibits FAO by engaging NEIL3, leading to the induction of anoikis in HCC. This study defines a pivotal role for NEIL3 in regulating anoikis in HCC and also offers initial proof-of-concept for Nilotinib as a therapeutic candidate. Subsequent investigations will employ HCC organoid systems and expanded clinical samples to comprehensively assess the efficacy and safety of targeting NEIL3 with Nilotinib in HCC. Furthermore, whether NEIL3 functions directly as a transcriptional co‑regulator by binding to the regulatory regions of FAO genes, or indirectly maintains the transcriptional environment of FAO genes entirely through its base excision repair activity, remains to be elucidated. Future studies employing chromatin immunoprecipitation sequencing (ChIP‑seq) and ATAC‑seq in NEIL3‑overexpressing and NEIL3‑knockdown cells could map its genome‑wide binding profile and chromatin accessibility landscape, thereby clarifying the direct genomic targets of NEIL3 and the downstream networks through which it regulates FAO gene expression.

## Data Availability

The original contributions presented in the study are included in the article/supplementary material. Further inquiries can be directed to the corresponding author.
